# A Review on the Life Cycle Assessment of Cellulose: From Properties to the Potential of Making It a Low Carbon Material

**DOI:** 10.3390/ma14040714

**Published:** 2021-02-03

**Authors:** Firoozeh Foroughi, Erfan Rezvani Ghomi, Fatemeh Morshedi Dehaghi, Ramadan Borayek, Seeram Ramakrishna

**Affiliations:** 1 Department of Materials Science and Engineering, Faculty of Engineering, National University of Singapore, 9 Engineering Drive 1, Singapore 117576, Singapore; ramadan_abdelhamid@u.nus.edu; 2 Center for Nanotechnology and Sustainability, Department of Mechanical Engineering, National University of Singapore, Singapore 117581, Singapore; morshedi.iut91@gmail.com

**Keywords:** nanocellulose, life cycle assessment, cradle-to-grave, low carbon materials, cradle-to-gate

## Abstract

The huge plastic production and plastic pollution are considered important global issues due to environmental aspects. One practical and efficient way to address them is to replace fossil-based plastics with natural-based materials, such as cellulose. The applications of different cellulose products have recently received increasing attention because of their desirable properties, such as biodegradability and sustainability. In this regard, the current study initially reviews cellulose products’ properties in three categories, including biopolymers based on the cellulose-derived monomer, cellulose fibers and their derivatives, and nanocellulose. The available life cycle assessments (LCA) for cellulose were comprehensively reviewed and classified at all the stages, including extraction of cellulose in various forms, manufacturing, usage, and disposal. Finally, due to the development of low-carbon materials in recent years and the importance of greenhouse gases (GHG) emissions, the proposed solutions to make cellulose a low carbon material were made. The optimization of the cellulose production process, such as the recovery of excessive solvents and using by-products as inputs for other processes, seem to be the most important step toward making it a low carbon material.

## 1. Introduction

Due to environmental issues caused by the consumption of fossil resources and their depletion, the concept of sustainable development using environmentally innocuous materials is being adopted [1,2,3]. Cellulose is the most abundant biodegradable polymer available, having a total production capacity of 10^11^–10^12^ tons each year [4]. The gross domestic product induced by the commercialization of nanocellulose, as a form of cellulose materials, is estimated to be around $600 billion worldwide by 2020 [5]. The U.S Department of Energy predicts that renewable sources will supply 50% of necessary chemicals by 2050 [6]. Therefore, research on different aspects of cellulose products, their application in various industries, and their production techniques have increased. Due to the increasing consumption of cellulose products in recent years, one of the required fields of this research, as well as the purpose of this study, is the evaluation of environmental effects arising from the extraction of cellulose, fabrication processes of cellulose products, cellulose products use, and their end-of-life disposal. In this regard, life cycle analysis (LCA) is a powerful tool for assessing the cumulative environmental impact attributed to all the steps from extraction-manufacture-use-dispose of cellulose, in other words, cradle to grave. Based on these analyses, measures can be taken to minimize the environmental impact and develop low-carbon cellulosic materials [7,8].

**Figure 1:**
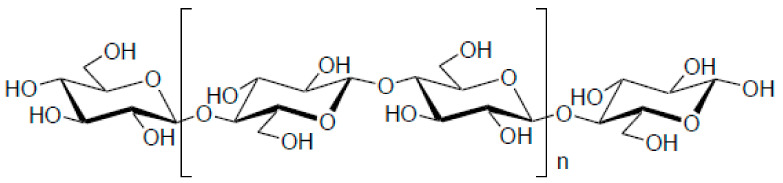
Structure of cellulose.

Payen first discovered cellulose in 1838, and its molecular formula was determined to be C_6_H_10_O_5_ by elemental analysis. The structure of this polymer is a linear syndiotactic homopolymer formed by D-anhydroglucopyranose units (AGUs), which are joined by glycosidic bonds (as shown in Figure 1) [9]. The primary source of cellulose is plants such as wood, hemp, cotton, and linen, and it has been used as an energy source and construction materials for thousands of years [10,11,12]. Moreover, cellulose is produced from many microorganisms such as fungi and bacteria having the same chemical makeup as was studied by Brown in 1886 [13,14]. Bacterial cellulose differs from a plant in a degree of polymerization, purity, and characteristics [10,15,16,17,18]. It is clear that due to having three hydroxyl groups in the monomer structure, the formation of hydrogen bonds has a noticeable influence on directing the crystalline regions and, as a result, controlling the physical properties [19,20]. The presence of these hydroxyl groups also eases cellulose’s chemical modification processes to obtain the cellulose derivatives that are useful for manufacturing new biopolymers with various applications [21,22,23].

In this study, different cellulose forms are first investigated in terms of characteristics, manufacturing processes, applications, and their effects on the final products. According to recent research, the environmental impacts of various forms of cellulose, including cradle-to-gate and cradle-to-grave, are analyzed through LCA. Finally, after highlighting the challenges faced by the LCA of cellulose materials, some specific suggestions related to the development of low-carbon materials are expressed.

## 2. Classification of Different Types of Cellulose

**Figure 2:**
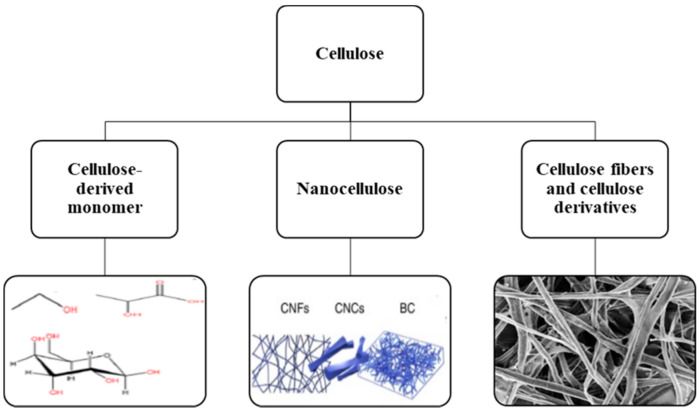
A general classification of different forms of cellulose.

Cellulose-based products are classified into three other groups based on the forms of cellulose, including (1) cellulose-derived monomer for biopolymers production, (2) products based on cellulose fiber and cellulose derivatives, and (3) products based on nanocellulose. Figure 2 shows three different cellulose forms, and their products, properties, and other applications are elaborated upon in the following sections.

### 2.1. Cellulose-Derived Monomer

Glucose can be obtained by depolymerizing cellulose via enzymatic and acid-catalyzed hydrolysis. Then, they are used for polymer production. Glucose monosaccharides act as raw materials for the catalytic and biotechnological production of chemical and monomers such as methanol, ethanol, lactic acid (LA), sorbitol, 5-hydroxymethylfurfural (5-HMF), levulinic acid (LevA). These derived monomers can be used to synthesize sustainable polymers and copolymers [24]. Table 1 lists some chemicals and polymers with their production routes as obtained by the depolymerization of cellulose.

### 2.2. Cellulose Fibers and Derivatives

In recent decades, cellulose fibers and cellulose derivatives have been widely used in the production of biocomposites and films as filler, matrix, or both. Cellulose plant fibers consist of a primary cell wall and three secondary cell walls. The cell walls are made up of bundles of microfibrils with a diameter in the order of 10 μm and are present in the lignin-hemicellulose matrix [35]. Many factors influence the properties of cellulose fibers, such as fiber structure, microfibrils angle, cell dimensions, and defects of plants, which differ for different plants and also parts of the plant [36]. Furthermore, chemical treatment is considered as a modification method of the plant fibers properties, which develops opportunities for new biopolymers with improved physical and chemical properties [37,38]. Among these derivatives, cellulose esters, ethers and acetate are the most common.

Recently, all-cellulose composites have emerged as a new class of biocomposites. These nanocomposites have received huge attention because of the use of chemically similar cellulosic materials as matrix and filler that can eliminate the problem of matrix-filler adhesion and interactions [39]. The cellulose used in these nanocomposites can be in the form of fibers or nanocellulose, which will be discussed in the next section. The results of using this form of cellulose in biocomposites and their subsequent applications are summarized in Table 2.

### 2.3. Nanocellulose

**Figure 3:**
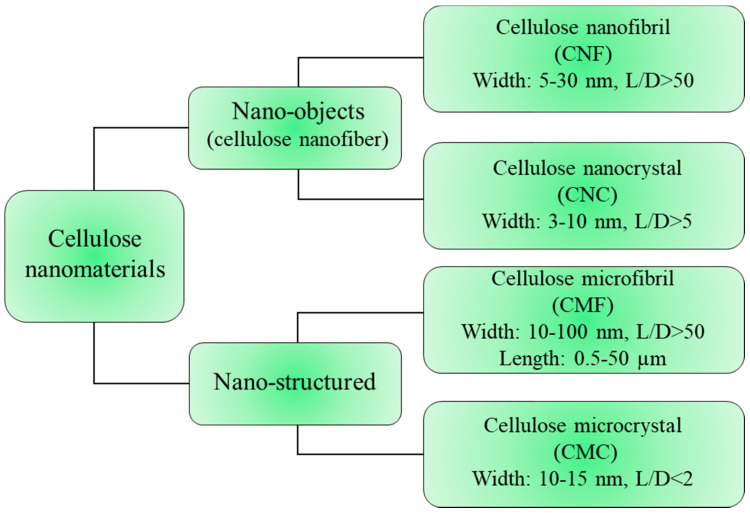
Classification of cellulose nanomaterials based on size and appearance.

In recent years, attention towards a novel form of cellulose is increasing considerably because of the attractive properties of nano-scale materials. There are some specific characteristics of nanocellulose, including excellent mechanical properties, high aspect ratio, and good biodegradability [24,56]. There are two different classifications for cellulose nanomaterials: a classification based on the size and appearance of nanocellulose, and the other is based on the preparation methods and raw materials [35]. Figure 3 illustrates the classification of cellulose nanomaterials based on size and appearance.

**Figure 4:**
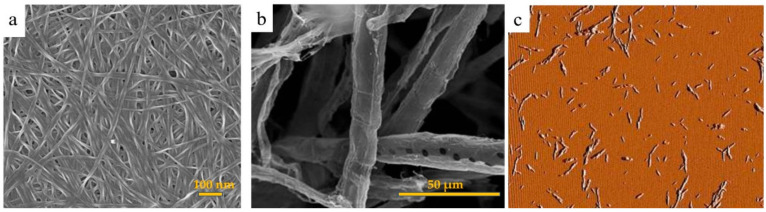
Scanning electron micrographs of (**a**) BNC [59], (**b**) Kraft bamboo pulp MFC [60], and (**c**) Atomic force microscopy (AFM) amplitude image of CNC (scan size: 5 μm or 5 by 5 microns) [61].

Furthermore, according to different preparation methods and raw materials, nanocellulose can be divided into three subcategories. This classification includes (1) cellulose microfibrils (CMF), or nanofibrils (CNF) prepared from plant fibers through chemical, mechanical, or combined treatments [57,58], (2) nanocrystalline cellulose (CNC) (sometimes called nanowhiskers) produced by raw materials including plant, animal, and bacterial resources through acid hydrolysis, and (3) bacterial nanocellulose (BNC) prepared from certain bacteria [5]. Figure 4 shows the images of these types of nanocellulose, and Table 3 provides some results and applications of these forms of nanocellulose.

## 3. Life Cycle Assessment (LCA) of Cellulose

**Figure 5:**
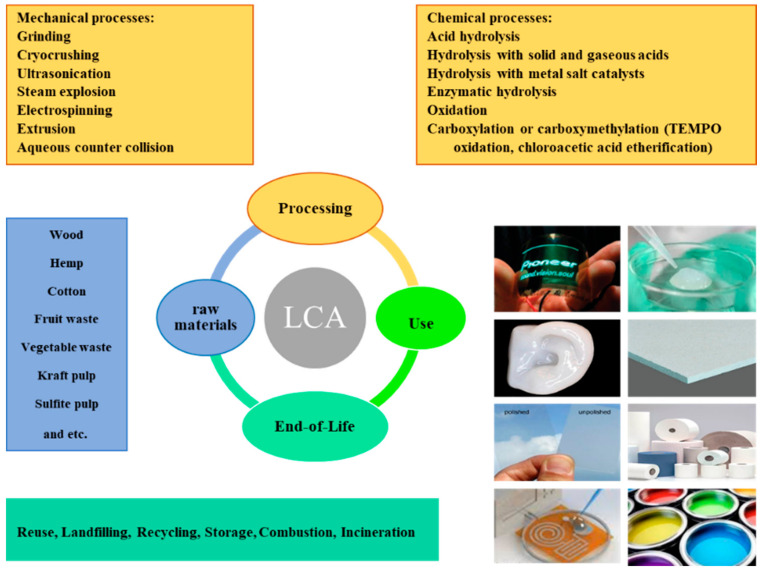
The life cycle of nanocellulose products, including raw materials, processing, use, and end-of-life stages.

LCA is a useful tool for assessing environmental impacts related to the extraction of raw material, manufacturing, use of final products, and disposal. In other words, all the stages from the fabrication of products by using raw materials (cradle) to end-of-life (EOL) disposal methods of final products (grave) constitute LCA. Since cellulose-derived monomers and their derivatives, the first and second groups, are converted to chemicals and other polymers, their LCA is not performed. Therefore, the LCA of nanocelluloses and their products will be elaborated on in the subsequent sections. Due to some challenges such as data unavailability, inconsideration towards end-of-life treatments, and limitations related to the lab-scale processes, there are only a few LCA studies related to cellulose products, i.e., cradle-to-grave. Therefore, we characterized our LCA studies into two categories of cradle-to-gate and cradle-to-grave; their detailed information is shown in Table 4. Figure 5 shows a schematic of all the stages of the life cycle of nanocellulose products. The LCA for each form of cellulose can be investigated in terms of cumulative energy demand (CED), ozone depletion (kg CFC-11 equivalents), terrestrial acidification (TA, kg SO_2_ equivalents), eutrophication (kg of phosphorus equivalent for freshwater, kg nitrogen equivalent for marine), water depletion (WD, cubic meters), human toxicity (HT, kg of 1,4-dichlorobenzene equivalent) and fossil fuel depletion (kg Oil equivalents), and climate change (CC) in the following sections. The evaluation of climate change in the life cycle, which is related to GHG emissions, resource depletion, global warming potential (GWP, kg CO_2_ equivalents), and waste generation, is done to develop low-carbon materials. The main tools that are used for assessing the environmental impacts include CED (SimaPro v1.08 software, PRé Sustainability, Amersfoort, The Netherlands), International Panel on Climate Change (IPCC) (World Meteorological Organization, Geneva, Switzerland) with a time frame of 100 years (IPCC7 GWP 100a v1.02), Eco-Indicator-99 (PRé Susteinability, Amersfoort, The Netherlands) (EI99, SimaPro v2.08 for human health, ecosystem quality and resources), CML2001 (the Institute of Environmental Sciences, Leiden, The Netherlands) and ReCiPe (endpoint or midpoint for different parameters) (RIVM, Utrecht, The Netherlands). In the ReCiPe method, the life cycle inventory results transform to a limited number of indicator scores, where each indicator score illustrates the relative severity of an environmental impact category. Eco-indicator 99 identifies 11 environmental impact categories into three environmental damages endpoints, including human health, ecosystem quality, and resources.

## 3.1. LCA from Cradle-To-Gate

Hohenthal et al. evaluated the environmental impact of CNF for the first time. Their study was based on the cradle-to-gate LCA for the production of one-ton CNF using sulfite pulp as raw material and three different processing routes shown in Table 4 and two laboratories and one pilot-scale study. LCA was performed using the ReCiPe method and included GWP, eutrophication, TA, water depletion, and fossil fuel depletion (Table 4) [81]. Besides, a difference in electricity consumption between chemical processes was investigated. Table 4 shows that there is a considerable difference in wastewater between three different processing routes. Moreover, enzymatic pre-treatment has more yield and consumes more energy amongst chemical pre-treatment processes. In contrast, the TEMPO oxidation reaction’s energy consumption is less than other strategies and has a lower yield.

Li et al. studied the Cradle-to-gate LCA for 10 g of MFC from kraft pulp as raw materials on a laboratory scale. The fabrication processes included chemical treatment followed by mechanical techniques [90,91]. In this regard, chemical pre-treatments included 2,2,6,6-tetramethylpiperidine-1-oxyl (TEMPO) oxidation and chloroacetic acid etherification [92,93,94,95]. Additionally, homogenization and sonication processes were selected as mechanical disintegration processes [90,91,96]. For samples treated by the sonication process, a centrifuge purifying process was required. Therefore, there were four possible routes of MFC production. CED (with SimaPro v1.08 software) and GWP (IPCC 7 GWP 100a v1.02) were assessed as two important environmental impacts. Furthermore, three main categories of environmental impacts, including human health, ecosystem quality, and resources, were assessed by Eco-Indicator 99 (EI 99, SimaPro v2.08) method and different egalitarian (E), hierarchist (H), and individualist (I) perspectives, which are long, medium, and short time horizon, respectively. The obtained results for CED and GWP are summarized in Table 4 [5]. Generally, they observed that the chloroacetic acid etherification and sonication processes require more energy for chemical and mechanical processes, respectively. Therefore, TOHO and CESO processes require a minimum and maximum energy, respectively. Besides, the GWP trend is similar to CED because CO_2_ is emitted by using fossil fuels. The results of EI 99 show that human health has more importance in short- term perspective, while resources become more important for hierarchist and egalitarian perspectives. It is worth mentioning that ecosystem quality stays the same factor for all three perspectives. Moreover, among different nanocellulose production methods, the TOHO and CESO routes have the lowest and highest environmental impacts in each perspective.

In another study performed by Piccinno et al., the environmental impact for cradle-to-gate LCA of 1 g CNF was evaluated. The authors extracted CNF from waste carrot (carrot or carrot pomace) and considered three routes for fabricating CNF in the laboratory (as explained in Table 4). In this study, the impact assessment’s different scenarios included GWP, CED, ecosystem quality, human health, and resources by ReCiPe midpoint and endpoint indicators with the hierarchist perspective [82]. The results of ReCiPe endpoint indicators of three routes show that electrospinning has a higher impact on the environment than wet spinning due to the smaller scale, lower yield (60%), and mainly the high-energy consumption during this process. In wet spinning (route 1a and 1b), the liberation of MFC was considered as the most energy-consuming stage. A closer look at the MFC liberation stage shows that the enzymatic treatment is the main contributor to environmental impact because a lot of energy is needed for heating and stirring the mixture at 40 °C for 24 h. Besides, this stage has the highest share of wastewater. On the other hand, according to the results of ReCiPe midpoint indicators for route 1a, the liberation of the MFC has the highest environmental impact. Besides, acetone usage in the solvent exchange and GripX production has a high potential for photochemical oxidant formation and terrestrial ecotoxicity, respectively.

In comparison with the production of 10 g of MFC, which was studied by Li et al., the total energy consumption for the enzymatic treatment was lower than HO and SO processes in Li et al. study because, in the wood pulp production process, chemicals were responsible for a significant portion of CED, while in this process, electricity contributes to about 95% of the CED. About GWP, the impact of the TOHO route (1.9 kg CO_2_ eq) was close to the process of this study.

In another study by Piccinno et al. on the impact assessment of CNF, the authors applied the scale-up framework to address the limitations of the lab-scale processes. In this regard, the authors only evaluated the Cradle-to-gate LCA for 1 kg of spun yarn process with the GripX coating (route 1: MFC liberated (enzymatic + homogenization) + coating MFC with GripX + wet spinning by adding sodium alginate). They examined different systems resulting in several scenarios, including different types of starting materials (carrot, carrot pomace), enzyme deactivation procedures (with heat or with a bleaching agent, ClO2), with or without heat, and solvent recovery, solvent exchange, or drying of the acetone. The authors evaluated the LCA of these routes based on depletion of resources, damage to human health, and ecosystem quality by applying the ReCiPe method (endpoint and midpoint) with the hierarchist perspective. The findings related to these scenarios show that: (1) using carrot pomace as a starting material reduces every step, such as transport, (2) using bleaching agent for deactivating the enzymes is preferable than heat deactivation, (3) solvent recovery in producing GripX has a considerable advantage [83].

In another study by Arvidsson et al., Cradle-to-gate LCA for 1 kg of CNF manufactured by wood pulp was studied by three different production methods (as shown in Table 4). For the manufacture of CNF, four different types of pulp were used, which contain elementary chlorine-free sulfate (ECF), totally chlorine-free sulfate (TCF), unbleached sulfate, and chlorine-bleached sulfite pulp. The environmental impact was studied in CED, GWP, TA, and WD, using the CED (SimaPro v1.08 software) ReCiPe method. The results of these categories are summarized in Table 4 [84]. According to Table 4, the environmental impact of the carboxymethylation route is significantly higher than other routes. To be more specific, for the carboxymethylation route, CED is higher compared to other routes because of the use of chemicals such as ethanol, isopropanol, and methanol. Besides, the pretreatment stage was the main contributor in GWP, TA, and WD. For the enzymatic route, pulp production has the main share in CED and GWP; while water usage in the washing stage and phosphate production, enzymatic treatment has more impact in WD and TA. The treatment process contributes more CED, WD, and GWP than pulp production for the no pretreatment route. However, the share of pulp production in TA is more considerable than in the treatment process.

In comparison with the study by Li et al., generally, the environmental impacts from the enzymatic and no pretreatment methods are lower than that of the TOHO process, which has the lowest environmental impact in that study.

**Figure 6:**
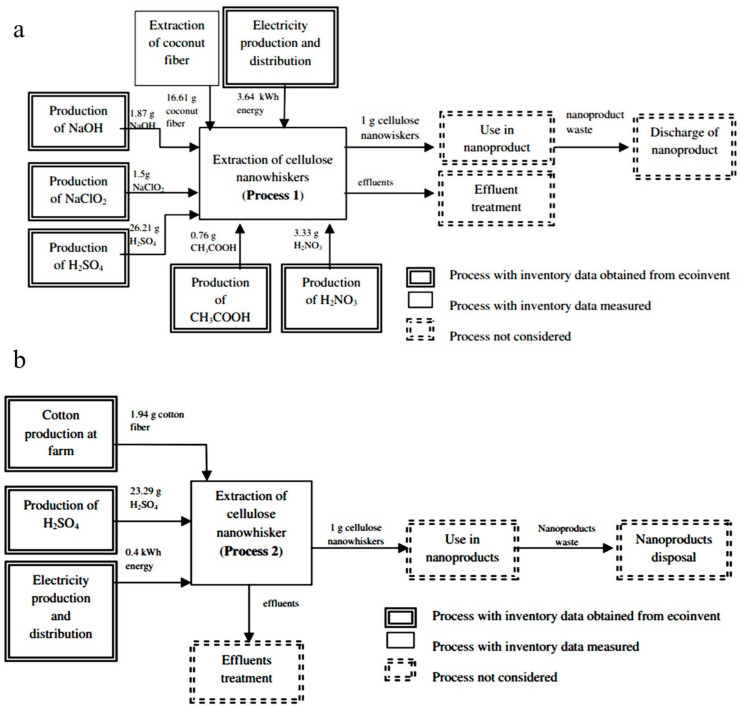
(**a**) EUC system (raw material: unripe coconut fiber), (**b**) EC system (raw material: cotton fiber) [85].

Figueiredo et al. studied the Cradle-to-gate LCA of 1 g CNCs for the first time. The authors produced CNCs through acid hydrolysis from two different raw materials, namely, unripe coconut fibers (EUC process) and white cotton fibers (EC process) on a laboratory scale. Figure 6a,b presents the system boundary for EUC and EC processes, respectively. The authors evaluated the environmental impact, including CC, WD, HT, and eutrophication, using the ReCiPe method. In the ReCiPe method, climate change expresses the results according to the IPCC. The obtained values for each parameter are summarized in Table 4. The EUC system considers extra environmental impact than EC system except for WD. To elucidate, water consumption in turbines at hydropower plants to produce energy in the systems is the main contributor to WD. Furthermore, due to copper’s use in cables that distribute electricity, the EUC system generates more toxic substances and nutrients, leading to human toxicity and freshwater and marine eutrophication. In the EC system, the production of cotton on farms has a significant share in eutrophication.

Additionally, the energy demanded by two process routes were compared with each other and other nanomaterials such as CNTs and carbon nanofibers. It is observed that the EUC system also demands more energy than the EC system. The extraction process in both systems is the main contributor to energy consumption. Compared to the production of 1 g carbon nanofibers, the production of 1 g CNC in the EC system has a lower impact on CC and HT, whereas the EUC system is affected at the same level [85]. The energy demand to make 1 g nanowhiskers in the EC system is lower than 1 g carbon nanotube and carbon nanofiber.

In another study of CNC production, Nascimento et al. evaluated the Cradle-to-gate LCA to produce 1 g of CNC from four different CNC extraction methods after extraction of coconut fiber on a lab-scale (as shown in Table 4). These methods were applied for recovering lignin by four other chemicals used to hydrolyze cellulose. Categories consisted of CC, TA, eutrophication (FE and ME), HT and WD were evaluated as environmental impact criteria via the ReCiPe method (at the midpoint level) with a hierarchical version. The results related to these assessments are summarized in Table 4 [86,87]. These results show that the CNU route has the lowest resource consumption and emission loads for producing 1 g CNC. Besides, this route has the highest yield among all the other routes. The usage of a high concentration of H_2_SO_4_ to speed up the hydrolysis of the amorphous domains leads to a decrease in the yield in the CNH2 method. Moreover, high reaction time, high demand for equipment use, and high selectivity of ammonium persulphate are the main reasons for the lower yield of the CNO method. However, the fabrication of CNC from the CNU route has more environmental impacts than the fabrication of CNF studied by Arvidsson et al. [84] and Piccinno et al. [83].

## 3.2. LCA from Cradle-to-Grave

**Figure 7:**
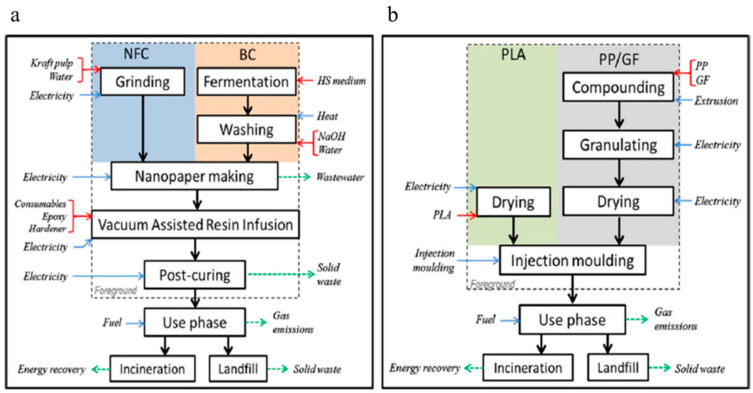
System boundaries for the life cycle of the (**a**) epoxy composites containing BC and CNF, (**b**) pure PLA and GF/PP composites. Red arrow: raw material required, blue: energy consumption, green arrow: material and energy wastes [88].

To complete the investigation of environmental impact, it is essential to evaluate all stages from the extraction of raw materials (cradle) to EOL of cellulose products (grave). In this regard, Hervy et al. studied cradle-to-gate and cradle-to-grave LCA of the epoxy/BC and CNF composites for the first time. The authors used PLA and reinforced polypropylene/30 wt% glass fiber (GF/PP) as benchmark materials for comparison. Figure 7 shows the system boundaries for the epoxy/BC and CNF composites’ life cycle, neat PLA, and GF/PP composites. Final products were considered to be used as automobile parts. Depending on the waste density, landfills, incineration to recover energy, and recycling were selected as the end-of-life treatments for the plastic wastes. GWP and abiotic depletion of fossil fuels (ADF) were used to assess the environmental impact via the CML2001 (April 2013 version) method developed by the Centre for Environmental Science in Leiden University.

Considering the results in Table 4, for BC/EP composites, ADF was more than pure PLA and GF/PP composites. However, their study showed that the amount of BC/EP composites required were less due to the higher tensile modulus. Besides, BC/EP composites have the highest GWP compared with other materials. On the other hand, CNF/EP composites showed the same results compared to pure PLA and GF/PP composites, except that their values were less than BC/EP composites. The reason should be the higher amount of CNF needed to reinforce the epoxy.

Generally, although the evaluation of cradle-to-gate LCA of epoxy composites containing BC and CNF shows higher environmental impacts than pure PLA and GF/PP, the cradle-to-grave LCA of the composite containing 60 vol.% nanocellulose was lower than that of the pure PLA and GF/PP composites. To be more specific, in comparison with neat PLA and GF/PP, neat PLA has a higher GWP among all composites. In contrast, GF/PP composites have the lowest values. The cause attributed to these results is that the mass required to achieve performance is less for GF/PP composites.

## 4. Challenges in Life Cycle Assessments (LCAs) of Cellulose Products

Studying the life cycle of cellulose products has become important because of greater attention paid towards the development of sustainable and renewable biopolymers. Life cycle assessment can be used as an effective tool for evaluating environmental impacts. However, since only laboratory data are available, there is no accurate assessment that includes all the impacts of cellulose products on different environmental aspects such as air emissions, human health, and waste stream discharges. On the other hand, data unavailability is one of the other limitations of lab-scale processes because they do not account for the end-of-life stage of the cellulose materials. At present, most studies conducted on the environmental impacts are related to the cradle-to-gate LCA [97,98].

In this view, scaling up of the production processes is one of the greatest challenges because, in many cases, laboratory processes are completely different from industrial processes. In the laboratory scale, subsequent steps are linked to each other regardless of material and energy recovery. On the other hand, the simple design of plants and considering the linear scaling factor for scale-up is not reasonable. For example, in Piccinno et al. study, the authors eliminated two CNF production routes due to the limitations of processing techniques. Besides, the authors considered several processes for recovering materials and heat [83].

## 5. Specific Strategies for Reduction of Energy Consumption and Development of Low-Carbon Materials

In the previous section, the LCA of cellulose materials was investigated by various criteria. In every step of the cellulose products’ life cycle, there are some issues related to the energy demand and GHG emissions, which should be addressed by technology developers and LCA practitioners. Some of these issues are associated with the stages from the extraction of cellulose to delivery of cellulose products and the use or service stage of cellulose products. There are insufficient findings in many cases, especially regarding the impact of starting material’s use on supply chains of other products, characterization of produced by-products and waste streams, and their impacts on supply chains of other products. Moreover, in the cellulose products’ end-of-life stage, information about disposal or recycling, the amounts of waste materials, and the release of emissions into the atmosphere are not specified. Considering the issues mentioned earlier, Table 5 shows some suggested strategies related to reducing energy consumption and developing low-carbon materials for future studies.

## 6. Conclusions

Considering the increasing consumption of cellulose products in recent decades due to their sustainable and biodegradable properties, this study deals with different aspects of this biopolymer. In this regard, the study initially reviews the properties of different cellulose forms, including cellulose-derived monomers and chemicals, cellulose fibers and their derivatives, and nanocelluloses. Then, some of the effects of these forms on manufactured products and their applications in diverse industries such as packaging, biosensors, and medicine were expressed.

Due to the importance of the impacts of various products on the environment, this study’s primary purpose is allocated to investigating the life cycle of cellulose materials and environmental impacts from the extraction of raw materials to the end-of-life of products. According to this, since two forms of cellulose, i.e., cellulose-derived monomers and cellulose derivatives, are converted to other chemicals and biopolymers, in this study only the life cycle of nanocelluloses was evaluated in two cradle-to-gate and cradle-to-grave categories. These assessments were performed with criteria including energy consumption and climate change containing global warming potential, eutrophication, terrestrial acidification, human toxicity, water depletion, and fossil fuel depletion. There are two significant challenges related to the evaluation of the cellulose products’ life cycle. The first challenge is data unavailability, especially in the use and disposal stages of nanocellulose products. In other words, many studies in this field assess the environmental impacts of cradle-to-gate. In addition, since most studies were performed on a laboratory scale, the results’ accuracy is still unknown. Given that nowadays, economic and climate change issues have received much attention from human societies, reducing the energy-intensive processes and development of low-carbon materials has become especially important. In this regard, in the final section of this study, some strategies related to achieving these goals in different manufacturing stages of the cellulose products have been suggested.

## Figures and Tables

**Table 1:** Chemicals and monomer/polymer-based on cellulose and their processes.

Cellulose-Derived Monomer	Process	Polymer/Chemical Materials	Process	Ref.
Ethanol	Conversion to ethanol by fermentation	Ethylene, Polyethylene, polyethylene oxide, polyvinyl chloride, polystyrene	Production of ethylene by ethanol dehydration at high temperatures	[25,26]
Sorbitol or sugar alcohols	Chemocatalytic transformations of glucose: hydrogenation or direct hydrogenolysis	Sorbitol, Isosorbide (base of polymers such as polyesters, polyamides, polycarbonates, polyurethanes, etc.), glycerol, propylene, ethylene glycol	Dehydration hydrogenolysis or hydrodeoxygenation reactions	[27]
LA	Fermentation of sugars	(1) Acrylic acid, 2,3-pentanedione, acetaldehyde, and propionic acid (2) propylene glycol, polycarbonates, polyurethanes, and polypropylene oxide) or pyruvic acid (3) alkyl lactates (4) PLA	(1) Dehydration of LA combined with other reactions (2) Reduction and oxidation (3) Esterification (4) Direct polycondensation, ring-opening polymerization	[26,28,29]
5-HMF	Acid-catalyzed dehydration of glucose	Furan-based monomers such as BHF and FDC	FDC: oxidation of HMF BHF: hydrogenation by NaBH4 or catalytic process by hydrogen over Cu or Pt	[26,30,31]
LevA	−	(1) γMMBL, βMMBL which are substitutes for the petroleum-based methacrylate monomers (2) constituents of PHA: 3HV, 4HV and their copolymers such as PHBV composites	(1) Two steps process developed by DuPont, condensation of 3-methyl-γ-butyrolactone (2) Conversion to 3HV and 4HV by microorganisms	[32,33,34]
Itaconic acid	Fermentation of glucose by fungi	New polyester based on poly (itaconic acid), polyamide	Transformation into itaconic diamide or 2-methyl-1,4-butanediamine	[31]

LA: Lactic acid, PLA: Polylactic acid, FDC: 2,5-furan dicarboxylic acid, BHF: 2,5-bis(hydroxymethyl)furan, 3HV: 3-hydroxyvalerate, 4HV: 4-hydroxyvalerate, LevA: levulinic acid, 5-HMF: 5-hydroxymethylfurfural, γMMBL: γ-methyl-α-methylene-γ-butyrolactone, βMMBL: β-methyl-α-methylene-γ-butyrolactone.

**Table 2:** The effects and applications of cellulose fibers and their derivatives.

Cellulose-Based Composites	Function of Cellulose	Results	Application	Ref.
BiOBr/regenerated cellulose fibers composite film	Matrix	Exhibiting efficient photocatalytic activity by providing a cavity for particles and extending the specific surface area.	A green catalyst for light degradation of phenol	[40]
Graphene Oxide (GO)/cellulose fibers composite film	Matrix	Improving mechanical properties: tensile strength (78%), elongation at break (172%), and fracture energy (397%) of composite film containing 0.5 wt% GO in comparison with the neat cellulose film. Excellent ultraviolet-shielding properties.	Packaging and protective industry	[41]
MWCNT/regenerated cellulose fibers film	Matrix	Improvement of ductility and toughness due to the favorable interaction of CNT-cellulose Enhancing thermal stability Effective sensing capability to tensile strain, temperature, and environmental humidity.	Biotechnological applications	[42]
Carbon nanotube (CNT)/cellulose fibers composite papers	Matrix	Enhancing electrical properties and thermal stabilities Ability to absorb microwaves in the range of 10.5 GHz.	Electromagnetic shields and biotechnological applications	[43]
Cellulose acetate/Hydroxyapatite composite	Matrix	Having uniform film and good ductility Adsorbing bisphenol A from polluted water Existence of strong interaction between CA and Hap.	Absorption of bisphenol A	[44]
Cellulose fiber/high-density polyethylene composite	Filler	Improvement in thermal and mechanical properties compared with neat HDPEIncrease of Young modulus by 122.4% in the composite containing 40 wt% cellulose fiber.	−	[45]
Polypyrrole/cellulose fiber composite	Filler	Reduction in highly toxic Cr(IV) to less toxic Cr(III).	Cr (VI) detoxification of contaminated water	[46]
Polypropylene/cellulose fiber composite	filler	Obtaining the best result of flame retardancy for silylation treatment of cellulose fibers.	Flame retardant composites	[47]
Cellulose lyocell fibers/cellulose acetate butyrate composite	Matrix/filler	Increase in Young modulus for composite containing 34.8 (*v/v*) (4 GPa) compared with cellulose acetate butyrate (2 GPa) Increase in tensile strength, water absorption, and biodegradability.	Water absorption	[48]
Regenerated cellulose/Cellulose nanowhiskers film composite	Matrix/filler	Having transparent to visible light film nanocomposites Improvement in mechanical properties, for example, tensile strength and modulus, could reach 124 MPa and 5 GPa.	Biomaterials and food ingredients	[49]
Regenerated cellulose/pulp fibres composites	Matrix/filler	A two-fold increase in tensile strength and elastic modulus in comparison with epoxy-cellulose composites.	−	[50]
All-Cellulose composites containing microcrystalline	Matrix/filler	Improving mechanical properties, e.g., tensile strength tensile modulus.	−	[51]
All-Cellulose composites containing bacterial cellulose	Matrix/filler	Providing a high toughness of 16 MJ/m^3^ Nanocomposites with optimum immersion time have a tensile strength of 411 MPa and a Young modulus of 18 GPa.	−	[52]
All-Cellulose aerogels containing microcrystalline	Matrix/filler	High mechanical properties, for example, for aerocellulose containing 10–15% cellulose, flexural strength and stiffness reached 8.1 MPa and 280 MP, respectively.	−	[53]
Cellulose diacetate/nanofibrillated cellulose film nanocomposites	Matrix/filler	Increase in tensile strength, Young modulus, and strain at the break by 102, 80, and 32%, respectively, with the addition of 15% nanofibrillated cellulose. Reduction in thermal stability and transparency by incorporation of nanofibrillated cellulose.	High-performance nanocomposites	[54]
All-Cellulose composites containing alpha and wood pulps fibers	Matrix/filler	By the addition of 38–50 wt.% of reinforcing fibers, an increase to 4 GPa and 14–16 MPa for Young modulus and yield stress was observed, respectively.	−	[55]

**Table 3:** Effects and applications of different forms of nanocellulose.

Type of Nanocellulose	Other Components	Results	Product Forms/Application	Ref.
CNF	Zeolites, polyethylene glycol, CaCl_2_	Improving tensile strength by 10 MPa Enhancing bending flexibility Removing thiols below the human olfactory threshold.	Film/air purification for odor removal	[62]
CNF	3-mercaptopropyl-trimethoxysilane	Showing high mechanical and chemical stabilities Recovering 94% of its shape in the water.	Sponge/adsorption of copper	[63]
CNF	Silica nanoparticles	A slight reduction in tenacity and preservation of Young’s modulusEnhancing char formation due to the presence of SNP on the CNF surface.	Fiber/fire retardants	[64]
CNF	−	Promising new piezoelectric material for sensors and actuators.	Film/piezoelectric sensor	[65]
CNF	Chitosan	Having higher piezoelectric response (7–8 pC/N) for solvent cast films based on CNF rather than CNC, chitosan and their blends Enhancing the flexibility by adding chitosan.	Film/piezoelectric sensor	[66]
CNF	Polyaniline	Showing proper conductivity because of the formation of continuous PANI coating over the CNFs. Attenuation of incoming microwave radiations via an absorption mechanism.	Composite nanopaper/supercapacitors, commercial electronic gadgets	[67]
CNF	Fe_3_O_4_ nanoparticles	Reduction in the mechanical properties due to the lower aspect ratio of added nanoparticles Avoiding the iron core in loudspeakers.	Membrane/loudspeakers	[68]
CNF	Polyethylene	Significant improvement in the cycling stability and safety of batteries based on Li metal Enhancement of wettability and thermal stability without shrinkage.	A tri-layer CNFs/PE/CNFs separator for lithium batteries	[69]
CNC	Chitosan, antibacterial agent	Reduction in air permeability by adding 8% CNC Enhancing paper resistance towards different microorganisms, specifically those causing food poisoning.	Paper sheet/food packaging	[70]
CNC	−	Color variation by exposure to NH_3_.H_2_O, H_2_O, HCl, acetic acid.	Coating/chemical sensors	[71]
CNC	Fluorophore tagged polystyrene latex bead	Having log_10_ reduction value (LRV) ≥ 6.3.	Membrane/removing of influenza virus	[72]
CNC functionalized with aldehyde groups	−	Proper adsorption of nisin and lysozyme onto the CNC.	Adsorption of lysozyme and nisin	[73]
Carboxylated CNC	Poly(N-isopropylacrylamide)	Exhibition the pH- and temperature- sensitivity Improving the stiffness by increasing the amount of CNC.	Hydrogel/biosensors	[74]
BC	Chitosan	Ability to retain moisture content for an extensive period without dehydration Showing a remarkable growth inhibition for Escherichia coli and Staphylococcus aureus Decreasing the tensile strength and elongation at break Increasing the Young modulus compared to BC No toxic effects on animal cells.	Membrane/wound healing	[75,76]
BC	PVA	Giving a broad range of mechanical properties, including a high degree of anisotropy.	Nanocomposites/Devices for replacing cardiovascular and other connective tissues	[77]
BC	gelatin	Having a proper interconnected pore network structure Improving thermal stability Having much better biocompatibility than pure BC by cell culture test.	Composites/Wound dressing, tissue-engineering scaffolds	[78]
BC	Platinum nanoparticle	Increasing thermal stability Showing high electro-catalytic activity.	Membrane/fuel cell and biosensor	[79]
Bacterial nano-cellulose	GO, Palladium	Showing highly efficient methylene orange degradation during filtration Removing contaminants including methylene blue, rhodamine.	Membrane/ultrafiltration	[80]

**Table 4:** A summary of LCA findings of nanocellulose products, including cumulative energy demand and climate change from cradle-to-gate.

Researchers/Type of Cellulose	Production Method	CED Value	GWP (kg CO_2_ eq)	ME/FE (kg N eq/kg p eq)	TA (kg SO_2_ eq)	Fossil Fuel Depletion (kg Oil eq)	Human Toxicity (kg 1,4-DB eq)	WD (kg or m^3^ H_2_O)	Key Assumption Made
Hohenthal et al./CNF [81]	Enzymatic + HPH	ــــ	1.2–3.1	0.015–0.016	0.008–0.045	0.3–0.75	ــــ	50	Enzymatic pretreatment has more yield and lower wastewater. Energy consumption of the TEMPO oxidation reaction is more in that process.
	TEMPO oxidation + HPH	ــــ	1.0–1.8	0.018–0.024	0.005–0.0065	0.25–0.5	ــــ	158	
	TEMPO oxidationn + mechanical refinement	ــــ	0.75–1.0	0.014–0.015	0.0045–0.005	0.20–0.25	ــــ	120	
Li et al./CMF [5]	TEMPO oxidation + Sonication + Centrifuge purifying (TOSO)	145.9 MJ	980 (per kg NC)	ــــ	ــــ	ــــ	ــــ	ــــ	Weight loss does not have a significant influence on LCA results. Both chemical modification processes (TO, CE) create similar anionic surfaces. The products of two mechanical disintegration processes (SO, HO) are the same. The batch processing capacity ratio of the HO process to the CE process is assumed three. Washing does not influence four different fabrication routes. Energy recovery of the incineration/combustion process was not considered because of complexity. Solvent evaporation was considered negligible.
	TEMPO oxidation + Homogenization (TOHO)	34.7 MJ	190 (per kg NC)	ــــ	ــــ	ــــ	ــــ	ــــ	
	Chloroacetic acid etherification + Sonication + Centrifuge purifying (CESO)	176.1 MJ	1160 (per kg NC)	ــــ	ــــ	ــــ	ــــ	ــــ	
	Chloroacetic acid etherification + Homogenization (CEHO)	64.9 MJ	360 (per kg NC)	ــــ	ــــ	ــــ	ــــ	ــــ	
Piccinno et al./CNF [82]	MFC liberated (Enzymatic + homogenization) + Coating MFC with GripX + Wet spinning by adding Sodium Alginate (route 1a)	32.2 MJ for production of 10 gr MFC	1.5–1.6 (10 g of MFC)	ــــ	ــــ	ــــ	ــــ	(0.201 for MFC liberation) 0.253 l/gr	All processes are performed in one place because of the lack of transport between the various partners.
	MFC liberated (Enzymatic + homogenization) + Wet spinning by adding Sodium Alginate (without coating) (route 1b)			ــــ	ــــ	ــــ	ــــ	(0.201 for MFC liberation) 0.255 l/g	
	MFC liberated (Enzymatic + homogenization) + electrospinning by adding PEO as a carrier polymer (route 2)			ــــ	ــــ	ــــ	ــــ	(0.201 for MFC liberation) 0.205 l/g	
Piccinno et al./CNF [83]		ــــ	ــــ	ــــ	ــــ	ــــ	ــــ	ــــ	ــــــــ
Arvidsson et al./CNF [84]	Enzymatic pretreatment+ microfluidization	87 MJ/kg	0.79	ــــ	0.0078	ــــ	ــــ	240	Neglecting the microbicide input due to low mass input toward produced CNF. The contribution of heat losses to the overall CED is neglected.
	Carboxymethylation pretreatment + microfluidization	1800 MJ/kg	99	ــــ	0.18	ــــ	ــــ	1000	
	Without pretreatment + homogenization treatment	240 MJ/kg	1.2	ــــ	0.0069	ــــ	ــــ	130	
Figueiredo et al./CNC [85]	EUC system	15.943 MJ for the extraction of raw materials	1.086412	0.000320/0.000134	ــــ	ــــ	0.291122	131 L/g	Transportation of coconut husks was not considered due to the installation of these units in the vicinity of companies extracting coconut water. The transportation of fibers and chemicals are neglected because of lab-scale processes.
	EC system	1.8 MJ for the extraction of raw materials	0.122171	0.000065/0.000024	ــــ	ــــ	0.034797	138 L/g	
Nascimento et al./CNC [86,87]	Extraction of CNC with dilute sulfuric acid (CNH1)	ــــ	ــــ	ــــ	ــــ	ــــ	ــــ	ــــ	Lignin was burned and used as a power source for cellulose nanocrystal extraction.
	Extraction of CNC with concentrated sulfuric acid (CNH2)	ــــ	ــــ	ــــ	ــــ	ــــ	ــــ	ــــ	
	Extraction of CNC with ammonium persulfate (CNO)	ــــ	ــــ	ــــ	ــــ	ــــ	ــــ	ــــ	
	Extraction of CNC with high powered ultrasound (CNU)	ــــ	0.207	5.68 × 10^−5^/3.03 × 10^−5^	0.00045	ــــ	0.0477	0.0023	
Hervy et al./BC/epoxy (BC/EP) and CNF/epoxy (CNF/EP) composites [88]	BC/EP CNF/EP	ــــ ــــ	~13.8 ~8.50	ــــ ــــ	ــــ ــــ	~270 MJ ~145 MJ	ــــ ــــ	ــــ ــــ	BC is produced by A.xylinum under certain conditions, which are specified in the reference [88]. Purification of BC also was performed in a specific state. The influence of additional epoxy resin during the process on the LCA results was not significant. Materials and energy losses during the processing of the epoxy/BC and CNF composites were assumed to be negligible. The environmental impacts related to transportation were disregarded. The energy requirement for fibrillating kraft pulp to CNF was determined according to the work of Josset et al. [89]. GaBi software was used for the production model of BC and CNF nano papers. The efficiency of all electrical appliances was assumed to be 100%. The durability of epoxy composites containing BC and CNF, PLA, and GF/PP composites were considered to be the same [88].

**Table 5:** Suggested strategies for reducing energy demand and development of a low-carbon economy for cellulose products.

Step of the LCA	Strategies for Reduction of Energy Demand	Strategies for the Development of Low-Carbon Materials
Extraction of raw materials	Usage of fruit and vegetable wastes reduces energy consumption [83].	Usage of fruit and vegetable wastes reduces environmental impacts [83].
Cellulose products manufacturing	Using better insulation and heat recovery during the process. Burning some by-products for using the generated energy [87]. Reducing solvent consumption can decrease energy consumption for the reaction indirectly. Reducing the reaction time can decrease power consumption [99].	Recovery of excessive solvents, especially in pretreatment reaction processes for reducing the environmental impact. Using by-products as inputs for other processes, specifically in large-scale processes [85]. Reusing of water used in the production of cellulose products, especially in the washing stage, for other processes [86].
Cellulose products use	−	Manufacturing of high-quality products increases the lifespan and decreases the environmental impacts.
Cellulose products end-of-life	−	Preventing the burning of wastes as much as possible due to the emission of toxic gases into the atmosphere.

## Data Availability

Data is contained within the article.
